# Temperature affects the repeatability of evolution in the microbial eukaryote *Tetrahymena thermophila*

**DOI:** 10.1002/ece3.8036

**Published:** 2021-08-23

**Authors:** Jason Tarkington, Rebecca A. Zufall

**Affiliations:** ^1^ Department of Biology and Biochemistry University of Houston Houston TX USA; ^2^ Department of Genetics Stanford University Stanford CA USA

**Keywords:** adaptation, convergence, correlated responses, experimental evolution, repeatability, temperature, *Tetrahymena*

## Abstract

Evolutionary biologists have long sought to understand what factors affect the repeatability of adaptive outcomes. To better understand the role of temperature in determining the repeatability of adaptive trajectories, we evolved populations of different genotypes of the ciliate *Tetrahymena thermophila* at low and high temperatures and followed changes in growth rate over 6,500 generations. As expected, growth rate increased with a decelerating rate for all populations; however, there were differences in the patterns of evolution at the two temperatures. The growth rates of the different genotypes tended to converge as evolution proceeded at both temperatures, but this convergence was quicker and more pronounced at the higher temperature. Additionally, over the first 4,000 generations we found greater repeatability of evolution, in terms of change in growth rate, among replicates of the same genotype at the higher temperature. Finally, we found limited evidence of trade‐offs in fitness between temperatures, and an asymmetry in the correlated responses, whereby evolution in a high temperature increases growth rate at the lower temperature significantly more than the reverse. These results demonstrate the importance of temperature in determining the repeatability of evolutionary trajectories for the eukaryotic microbe *Tetrahymena thermophila* and may provide clues to how temperature affects evolution more generally.

## Graphical abstract

A broad body of research has asked the question of when evolution can be repeatable. Here, we show the effect of temperature on the repeatability of evolutionary trajectories of replicate populations of the ciliate *Tetrahymena thermophila*. We also show that evolution temperature impacts the rate of convergence between divergent genotypes and the correlated responses at the alternate temperature.

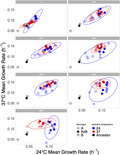

## 1 INTRODUCTION

The evolutionary trajectories of populations, both in the laboratory and in nature, are often remarkably similar to each other (Colosimo et al., 2005; Conte et al., 2012; Lenski & Travisano, 1994; Nosil et al., 2018; Woods et al., 2006). However, there can also be substantial differences in the trajectories of initially identical experimental populations (Blount et al., 2008) and populations in nature (Barluenga et al., 2006; Dieckmann & Doebeli, 1999; McKinnon & Rundle, 2002). While studies examining how population fitness changes through time have provided valuable insights into the repeatability of evolutionary trajectories, we still lack a comprehensive understanding of what conditions are likely to constrain trajectories from diverging due to stochastic forces, and thus contribute to the repeatability of evolution.

Previous work has demonstrated that temperature can fundamentally alter evolutionary outcomes, for example, by increasing biological diversity at lower latitudes (Allen et al., 2006; Gillooly et al., 2004; Roy et al., 2002). One purported explanation for the effect of temperature is that mutation rates are different at different temperatures. However, empirical results are mixed, with some results showing higher mutation rates at higher temperatures, others lower rates at higher temperatures, and yet others are inconclusive (Faberge & Beale, 1942; Ryan & Kiritani, 1959; Lindgren, 1972; Berger et al., 2017; Chu et al., 2018). Temperature could also affect adaptive outcomes indirectly by altering population densities and selective pressures. The “hotter is better” hypothesis predicts that warm‐adapted populations will have higher maximum performance than their cold‐adapted counterparts because thermodynamic constraints on reaction rates decrease with increasing temperature (Angilletta et al., 2010; Huey & Bennett, 1987). Evidence from comparative and experimental populations largely supports this hypothesis (e.g., Knies et al., 2009); however, again, some results are mixed (reviewed in Angilletta et al., 2010). Evidence from laboratory‐evolved *Escherichia coli* shows that greater fitness gains occur at higher temperatures and that populations evolved at lower temperatures show trade‐offs at higher temperatures but not vice versa (Bennett & Lenski, 1993; Mongold et al., 1996). Later work suggested that while the genetic changes underlying temperature adaptation were temperature specific, these mutations were also beneficial across all temperatures (Deatherage et al., 2017), demonstrating that the observed trade‐offs are not due to antagonistic pleiotropy. Overall these results demonstrate that temperature fundamentally affects adaptive outcomes, yet it remains unknown whether the temperature at which a population evolves will also affect the repeatability of adaptive trajectories.

To assess how temperature affects the repeatability of evolution, we performed a long‐term evolution experiment using the microbial eukaryote *Tetrahymena thermophila*. *T. thermophila* is useful as a model system due to its complex life history and development, and its ease of growth and tractability in laboratory (Merriam & Bruns, 1988; Nanney, 1974; Prescott, 1994). The short generation time and small cell size mean that large populations can be evolved over many generations in the laboratory, and population size and growth rate are easily monitored. In addition, in contrast to most other microbes in which experimental evolution is regularly performed, it has a complex life history and genome structure (Merriam & Bruns, 1988; Nanney, 1974), allowing us to test whether the general patterns found in other microbes, including prokaryotes, also apply to single‐celled eukaryotic ciliates.

*Tetrahymena thermophila*, like all ciliates, is notable for its genome structure. Two types of nuclei are maintained in each cell. The germline micronucleus (MIC) is diploid and transcriptionally silent during growth and asexual reproduction, while the somatic macronucleus (MAC) is 45‐ploid and transcriptionally active, meaning it gives rise to the phenotype of the cell (Merriam & Bruns, 1988). Ciliates are facultatively sexual, mostly reproducing asexually, but occasionally undergoing conjugative sex with cells of a different mating type (Nanney, 1974). Two features of the *T. thermophila* genome may potentially impact the patterns of adaptive evolution. First, the polyploid MAC divides by amitosis, a process that results in the random distribution of alleles among daughter cells. Unlike with division by mitosis, amitosis results in allelic variation among asexual progeny (Doerder et al., 1992), which generates higher levels of genetic variation and potentially increases the rate of evolution. Second, *Tetrahymena* has an exceptionally low base‐substitution mutation rate (Long et al., 2016), which has the potential to slow the rate of adaptation. However, the deleterious mutation rate is comparable to other species (Long et al., 2013), so the potential effect of mutation rate is currently unclear. *Tetrahymena thermophila* lives in freshwater lakes in the eastern United States and experiences a large range of temperatures throughout the year (Zufall et al., 2013). In the laboratory, strains are generally grown near their optimum temperature at ~30°C.

In this study, we conducted a long‐term evolution experiment to determine how temperature affects repeatability of evolution in a ciliate. We evolved populations of different genotypes of *T. thermophila* in two different temperatures (24 and 37°C) and monitored the fitness trajectories of replicate populations. To assess the effects of temperature on the dynamics of evolutionary trajectories, we asked: (a) Does the temperature at which populations evolve affect the future convergence or continued divergence of initial historical differences between genotypes, (b) does evolution temperature affect the repeatability of fitness trajectories, and (c) how temperature‐specific are adaptations, that is, are there trade‐offs or other correlated responses between temperatures? We hypothesized that temperature plays an important role in the way that variation is generated and acted on by selection. We therefore predicted that temperature would affect both the rate at which populations converge and the repeatability of evolution. Given prior results on trade‐offs (Bennett & Lenski, 1993; Deatherage et al., 2017; Mongold et al., 1996), we specifically predicted that populations evolving at a lower temperature would be more likely to experience trade‐offs.

## 2 METHODS

### 2.1 Summary

We evolved 24 populations in total—12 populations at 24°C and 12 populations at 37°C. Each set of 12 populations consisted of four replicate populations of three initial genotypes: two independent natural isolates and a hybrid progeny of these two isolates. Throughout the course of 6,500 generations of evolution, we measured growth rate at both 24 and 37°C for each population. 37°C is near the thermal maximum of *Tetrahymena*, while 24°C is well below the thermal optimum (~30°C). All populations contained only a single mating type, which prevented sexual reproduction during evolution, thus only mutations and existing variation in the MAC was subject to selection and captured in our fitness assays.

### 2.2 Strains and initial cross

Natural isolates of *T. thermophila*, designated 19617‐1 (which we refer to as A; *Tetrahymena* Stock Center ID SD03089; collected in Pennsylvania, USA; *cox1* GenBank: KY218380) and 19625‐2 (B; collected in Pennsylvania, USA; *cox1* GenBank: KY218383; Doerder, 2019), were thawed from frozen stocks, inoculated into 5.5 mL of the nutrient‐rich medium SSP (Gorovsky et al., 1975) in a 50‐mL conical tube, and incubated at 30°C with mixing for 2 days. These cultures were maintained as the parental lines. Eight populations were established for each genotype in 10‐mL cultures in SSP. Four of these were maintained at 24°C and four at 37°C. These populations were designated by genotype (A or B) – replicate (1–4) –evolution temperature (24 or 37°C), for example, A‐1–37.

To generate the hybrid genotype from these strains, a conical tube of each parental genotype was centrifuged and the supernatant was poured off before the cells were resuspended in 10 μM Tris buffer (Bruns & Brussard, 1974). After mixing at 30°C in Tris for two days to starve the cells and induce sexual competence, 1 mL of each starved parental population and an additional 1 mL of 10 μM Tris buffer were added to one well in a six‐well plate and placed back in the 30°C incubator. The next morning (~12 hr later) the plate was checked for pairs and put back in the incubator for an additional 4 hr to allow progression of conjugation. Individual mating pairs were isolated under a microscope using a 2‐μL micropipette and placed in 180 μL of SSP in one well of a 96‐well plate. The plate was then incubated for 48 hr after which time a single cell was isolated from each well and re‐cultured into 180 μL of fresh SSP in a new well. After another 48 hr at 30°C, four individual cells were isolated from one of the wells, into new wells with SSP, one for each of the replicate populations, and incubated at 30°C for 48 hr. Each of the four 180‐μL cultures was then split in two with each half being added to a separate 50‐mL conical tube containing 10 mL of SSP, one designated for evolution at 37°C and the other at 24°C. These eight cultures are the starting hybrid populations and are designated as A × B − replicate (1–4) – evolution temperature. The clonal nature of these hybrid populations means that they contain only a single mating type and further conjugation is not possible.

This provided us with a total of 24 populations consisting of three genotypes, two parental and one hybrid, half of which were evolved at 24°C and half at 37°C with four replicate populations of each genotype per treatment. Ancestral populations were frozen at the start of the experiment and periodically throughout; however, all growth assays were performed in real time and not on frozen samples.

### 2.3 Transfer regime

Approximately 25,000 cells (~90 μL) from each 37°C culture and 60,000 cells (~1 mL) from each 24°C culture were transferred to 10 mL of fresh SSP daily. Transfer volumes were adjusted as needed to maintain the same starting culture density at each transfer. On average, the 37°C evolved populations achieved ~6.8 generations per day and the 24°C populations achieved ~3.5 generations per day. This means that 37°C evolved populations experienced a wider range of densities during growth (~2,500–~275,000 cells/mL) than the 24°C evolved populations (~6,000–~60,000 cells/mL), starting with a lower density and ending at a higher density. We estimate the effective population size to be approximately 100,000 cells for each evolved environment by calculating the harmonic mean of the population size at each discrete generation (Karlin, 1968). To date, the 37°C populations have undergone ~12,000 generations of evolution and the 24°C populations have undergone ~6,500 generations of evolution. Here, we describe the changes in growth rate over the first 6,500 generations of evolution at each temperature.

### 2.4 Growth curves and analysis

As evolution progressed, growth rates of each population were measured at both 37°C and at 24°C, that is, at both the temperature at which they evolved and the alternate temperature, on average every ~10–30 generations. Variation in number of generations between measurements arose because we could not perform 37 and 24°C assays on the same days and the assays took different lengths of time at each temperature, and thus, we would typically do two consecutive single days of 37°C assays, followed by a single 24°C assay that lasted 2 days. Growth rate was measured by inoculating ~500–1,000 cells into one well of a 96‐well plate and measuring the optical density (OD) at 650 nm in a microplate reader every 5 min over the course of 24–48 hr for 37°C assays and 48–72 hr for 24°C assays (see below for validation of use of OD_650_ as a proxy for cell density). The maximum growth rate was then estimated for each well by fitting a linear regression to the steepest part of the growth curve (with OD on a log scale), estimating the maximum doublings per hour (h^−1^) (Long et al., 2013; Wang et al., 2012). 3–4 replicates of all populations were measured on a plate at each time point. ~600 plates containing 37°C evolved populations and ~850 plates containing 24°C evolved populations were run providing approximately 600–1,800 growth curves at either temperature per population over the 6,500 generations analyzed here.

### 2.5 Validation of optical density as proxy for cell density

To validate that OD accurately measures cell density over a range of densities, cells from cultures growing on the microplate reader were counted under the microscope at several points during the growth cycle. 3–4 replicate wells were inoculated, and the plate was run on the microplate reader at 37°C. Every two to three hours, 5 μl of culture was removed and at least 200 cells were counted to estimate cell density. The cells were diluted as needed and then counted in 10 μl droplets containing approximately 40 cells. This process was independently repeated two times. The cell density measured by counting was tested for correlation with the OD measured by the microplate reader at each time point, and OD was found to be a good indicator of cell density (Pearson's correlation coefficient = 0.9602).

### 2.6 Correlation of competitive fitness and growth rate

Because it is not technically feasible in this system to measure competitive fitness for the whole experiment, we measured the competitive fitness of a subset of the evolved lineages at one time point, after ~1,000 or ~3,500 generations (for populations evolved at 24 or 37°C, respectively) and compared this fitness metric to our measurements of growth rate. Competitive fitness was measured in replicate by competing a GFP labeled strain (Cui et al., 2006) against the experimental strain. The two strains were mixed in approximately 1:1 ratios, and the density of both strains was determined using a flow cytometer. The culture was allowed to grow overnight at room temperature after which time the flow cytometer was used again to measure the ratio of the two strains. Competitive fitness was calculated by dividing the natural log of the ratio of the final population density to the initial population density of one strain by the natural log of the ratio of the final population density to the initial population density of the other strain (Wiser & Lenski, 2015). Competitive fitness estimates correlated with our growth rate estimates (Pearson's correlation coefficient = 0.7999), indicating that growth rate is a good proxy for fitness.

### 2.7 Data analysis

~55,000 growth curves were collected from all populations over the first 6,500 generations of evolution, a period of over five years for the room temperature populations. This provided us with an average of more than 2,000 growth rate estimates per population over this period.

A generalized additive model (GAM) was fit to the mean growth rate of each population per plate (*R*_P_) assayed in the environment in which they evolved. Growth rate was fit as a function of generations (*N*), and models were fit that included various combinations of the terms genotype (*G*) and temperature (ET). The AICcs of each model were compared, using evidence ratios (ER = *e* ^(0.5*ΔAICc)^), to the full model including pairwise and three‐way interactions:

$$R_{P}∼N+G+\text{ET}+N*G+N*\text{ET}+G*\text{ET}+N*G*\text{ET}$$

The three‐way interaction term indicates whether the genotypes change differently at either temperature; that is, are there differences in the patterns of convergence or divergence among genotypes between the two temperatures? We also fit a standard least square model to the same dataset to calculate the scaled effects of each of the parameters from the best fit GAM model, that is, the full model.

We fit three different models to the growth rate trajectories of all populations assayed in the environment in which they evolved:

$$\text{Linear model}:R_{P}∼R_{0}+\Theta_{1}*N$$

$$\text{Power law model}:R_{P}∼R_{0}+\Theta_{2}*N^{\Theta 1}$$

$$\text{Hyperbolic model}:R_{P}∼R_{0}+\left(\Theta_{1}*N\right)/\left(\Theta_{2}+N\right).$$

where *R*_0_ is the mean growth rate of the ancestor and Θ_1_ and Θ_2_ are constants. We computed the AICc of each model and calculated the evidence ratio (ER = *e* ^(0.5*ΔAICc)^) to determine which best fit the evolutionary trajectory.

To assess specific time points, as well as for simplicity in visualization, growth rate data were binned into 250‐generation intervals for our repeatability analysis (generation 0 = 0–125, generation 250 = 125–375, generation 500 = 375–625, etc.) and 1,000‐generation intervals for other analyses where a finer level of resolution was not informative. The mean growth rate at both temperatures for each population was calculated for each bin. For each population, the 1,000‐generation bin with the highest growth rate for either temperature was identified and the mean absolute (i.e., maximum mean population growth rate in a 1,000‐generation bin minus the growth rate of the ancestor of that population) and relative increase (i.e., (absolute increase/ancestral growth rate) × 100) in growth rate was calculated from this with 95% confidence intervals (Table 1).

**TABLE 1:** (a) Mean maximum increase in absolute growth rate (h^−1^) and (b) mean maximum relative increase in growth rate for each genotype, evolution environment, and assay temperature with 95% confidence intervals. The overall mean absolute or relative increase of all 12 populations regardless of genotype is also shown

	Evolved at 24°C		Evolved at 37°C	
	Assayed at 24°C	Assayed at 37°C	Assayed at 24°C	Assayed at 37°C
(a)				
Genotype A	0.074 (0.054–0.095)	0.078 (0.057–0.098)	0.076 (0.067–0.084)	0.090 (0.069–0.112)
Genotype B	0.068 (0.064–0.073)	0.055 (0.026–0.084)	0.060 (0.042–0.078)	0.066 (0.058–0.074)
Genotype A × B	0.068 (0.060–0.076)	0.069 (0.054–0.084)	0.060 (0.053–0.068)	0.076 (0.066–0.086)
Overall	0.070 (0.065–0.075)	0.067 (0.057–0.077)	0.065 (0.059–0.072)	0.078 (0.069–0.086)
(b)				
Genotype A	353% (256%–451%)	160% (117%–202%)	359% (319%–400%)	186% (142%–230%)
Genotype B	152% (142%–162%)	67.1 (31.6%–102%)	134% (94.7%–174%)	80.3% (70.7%–90.0%)
Genotype A × B	157% (138%–175%)	93.7% (72.9%–115%)	140 (123%–157%)	104% (90.4%–117%)
Overall	221% (155%–286%)	107% (78.2%–135%)	211% (141%–282%)	123% (91.7%–155%)

ANOVAs were performed on these data to model the effects of genotype (*G*), assay temperature (AT), and evolution temperature (ET) on absolute or relative increase in growth rate (*R*_A_ or *R*_R_):

$$R_{A}\;\text{or}\;R_{R}∼G+\text{AT}+\text{ET}+G*\text{AT}+G*\text{ET}+\text{AT}*\text{ET}+G*\text{AT}*\text{ET}$$

For each ANOVA, the residuals were checked for heteroscedasticity both visually and by regression analysis and none was detected.

ANOVAs were also performed separately on the 48 data points (24 populations x 2 assay temperatures) from the 1,000‐generation bins at generations 0, 2,000, and 6,000 to test for the effect of assay temperature (AT), evolved temperature (ET), genotype (*G*), and their interactions as evolution progressed:

$$R_{B}∼G+\text{AT}+\text{ET}+G*\text{AT}+G*\text{ET}+\text{AT}*\text{ET}+G*\text{AT}*\text{ET}$$

where *R*_B_ is the growth rate in each bin. Pairwise *t*‐tests were also performed on each 1,000‐generation bin separately for each temperature and only including data from the evolution environment to assess whether the four replicates of one genotype are significantly different from the replicates of the other genotypes (Figure 2b).

### 2.8 Estimating repeatability

To test for differences among populations evolved from a single ancestor, that is, replicate populations of a single genotype, nested ANOVAs were performed on the first and the last 1,000‐generation bin:

$$R_{P}∼G+S\left[G\right]+\text{ET}+G*\text{ET}$$

where replicate (*S*) is nested within genotype and is treated as a random effect. These analyzes include only data collected at the evolution temperature.

To test for differences in the variance among replicate populations between evolution temperatures, ANOVAs were performed separately for each evolution temperature on the 250‐generation binned data:

$$R_{P}∼G+S\left[G\right]$$

where replicate (*S*) is nested within genotype and is treated as a random effect.

From this, variance components (which are scaled to the mean) attributable to replicate population were computed to assess the amount of variation that results from differences among replicate populations; the inverse of this was our measure of repeatability (Figure 3).

The same analysis was performed without nesting replicate population in genotype to assess the total variance among all populations as evolution progressed:

$$R_{P}∼S$$

where replicate (*S*) is a random effect (Figure 4). This analysis shows how the variation between replicates within a genotype combines with the variation that results from differences between genotypes. At each binned time point, Levene's tests were performed to assess whether the variation in growth rate among all populations is significantly different across temperatures.

## 3 RESULTS

### 3.1 General patterns of adaptation

**FIGURE 1:**
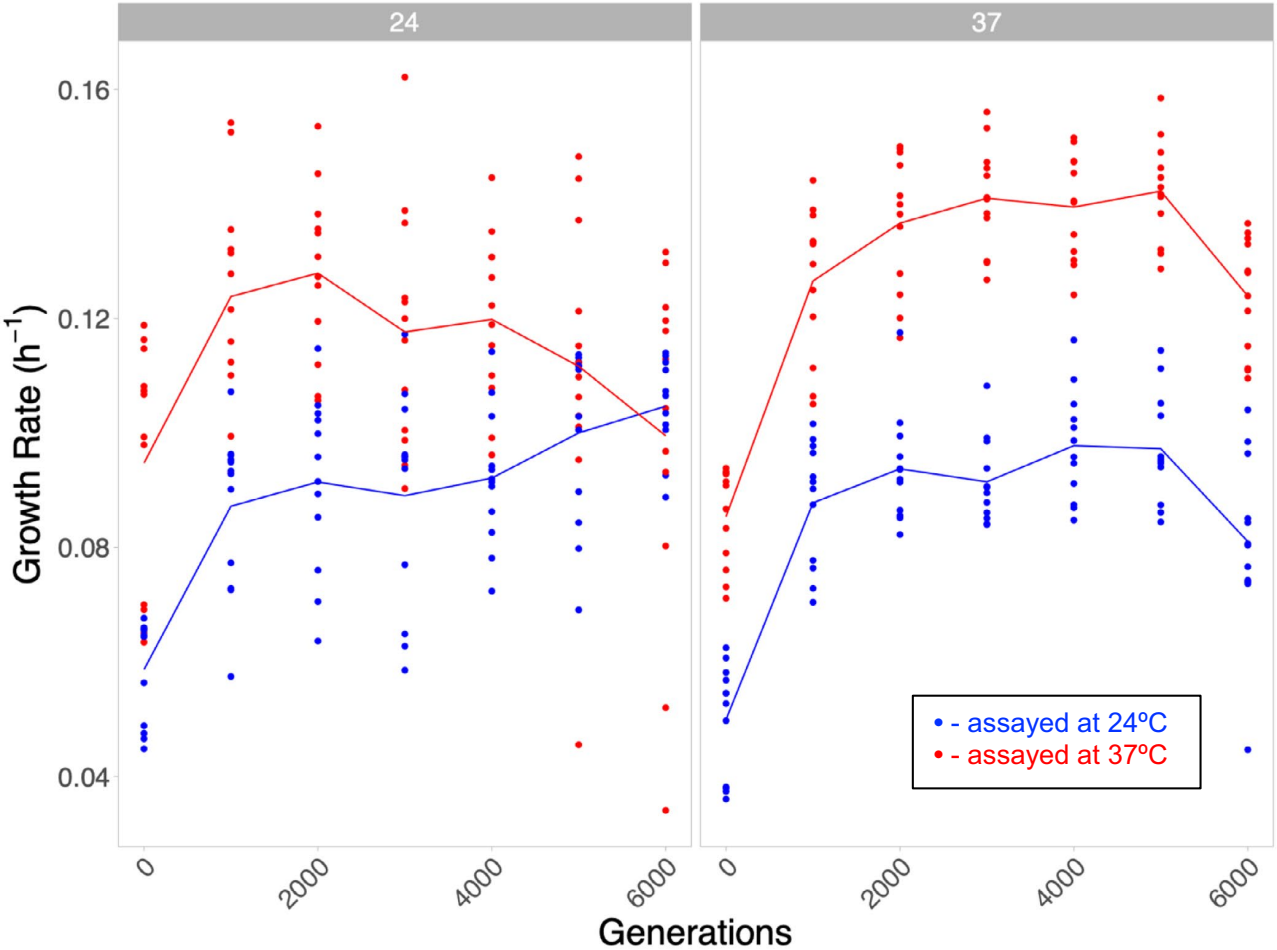
Overall pattern of evolution across all populations evolved at 24 and 37°C. Each point shows the growth rate of a population and the line indicates the mean growth rate of populations assayed at 24°C (blue) and 37°C (red) when evolved at 24°C (left panel) and 37°C (right panel) over 6,500 generations. Data are binned into 1,000 generation intervals, with the first bin containing generations 0–500

**FIGURE 2:**
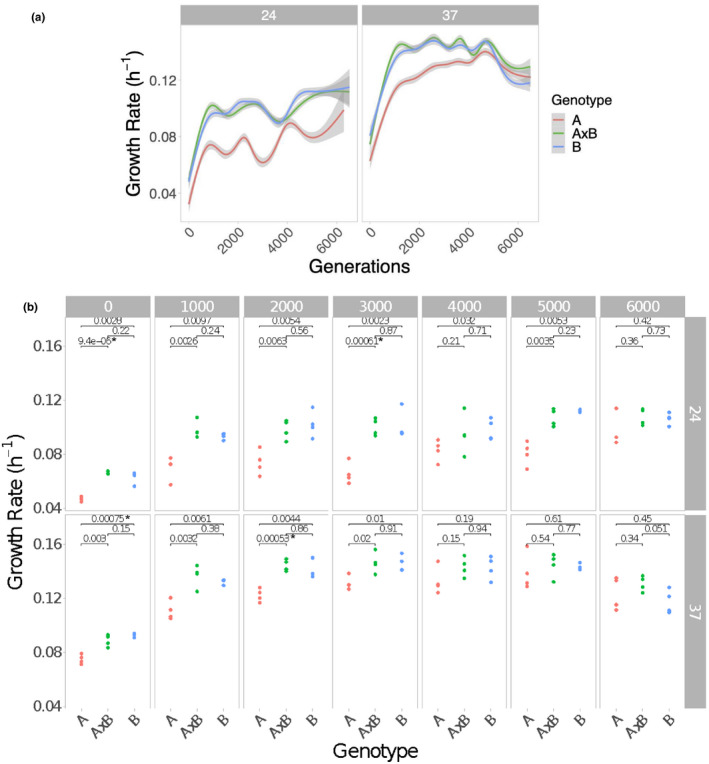
(a) Fitness trajectories of each genotype assayed in their evolved temperature. Smoothed curves produced using “gam” function in R. The shaded area shows the 95% confidence intervals of the four replicate populations for each genotype. The left panel shows populations evolved and assayed at 24°C and the right shows populations evolved and assayed at 37°C. The initially slower growing genotype (A) converges with the other two genotypes at 37°C but not at 24°C. (b) Genotypes converge on similar growth rates faster at the higher temperature. Differences in mean growth rates in the home environment (i.e., assay temperature the same as the evolution temperature) among genotypes (A = red, A × B = green, B = blue) are shown in 1,000‐generation bins at each temperature. Each point shows the mean growth rate of one out of the four replicate populations. A *t*‐test was used to determine significant differences between genotypes, and the p‐values are reported for each pairwise comparison. The Bonferroni corrected significance threshold for this analysis is 0.05/42 = 0.00119. Asterisks (*) indicate comparisons that pass the Bonferroni corrected significance threshold

All populations showed the expected pattern of increased growth rate over the course of the experiment. The trajectories of evolving laboratory populations often follow a pattern of a decelerating rate of return, characterized by larger fitness increases early in the experiment, followed by incrementally smaller increases in subsequent generations (Couce & Tenaillon, 2015; Schoustra et al., 2016; Wünsche et al., 2017). Our results follow this pattern (with a linear model fitting the trajectories poorly) at both temperatures (Figure 1) and in all three genotypes (Figure 2a), suggesting that experimental evolution in the ciliate *T. thermophila* does not fundamentally differ from other taxa.

At the start of the experiment, there was a significant difference in growth rate between genotypes (ANOVA on *R*_B_ in bin 0: *F*(2,38) = 87.6, *p* < .0001, Bonferroni corrected significance threshold = 0.00714) at both assay temperatures (Figure 2b). Specifically, one of the parental genotypes (A) grew slower than the other parental genotype (B) and the hybrid genotype (A × B) at both temperatures. Previous experiments have shown that populations founded by initially slower growing genotypes tend to increase more in growth rate over the course of an experiment than those founded by initially faster growing genotypes (Jerison et al., 2017; Wünsche et al., 2017). We found a qualitatively similar result whereby genotype had a significant effect on the absolute increase (ANOVA on *R*_A_: *F*(2,38) = 12.67, *p* < .0001, Bonferroni corrected significance threshold = 0.00714) and the relative increase (ANOVA on *R*_R_: *F*(2,38) = 197.55, *p* < .0001, Bonferroni corrected significance threshold = 0.00714) in growth rate. Populations founded by the slowest growing genotype (A) experienced the largest increases in growth rate for all four combinations of evolution temperature and assay temperature (Table 1). However, due to the small number of genotypes we cannot definitively say this effect is due to the initially lower growth rate of genotype A.

Unlike the long‐term evolved *E. coli* lines, which continue to increase in fitness even after 60,000 generations (Lenski et al., 2015), we find no significant change in mean growth rate among populations over the last 2,000 generations of evolution; in fact, our estimate of mean growth rate drops slightly from 0.1159 divisions per hour (h^−1^) in the 4,000‐generation bin to 0.1147 hr^−1^ in the 6,000‐generation bin. Additionally, a hyperbolic model yields a substantially better fit than a power law model or a linear model (AIC evidence ratio; AIC value hyperbolic: −38788.3, power law: −38406.61, linear: −37689.79). This result was consistent across temperatures. This suggests that the populations may have reached growth rate optima upon which further improvement is unlikely. However, given the limited number of generations and smaller population sizes, we are cautious in interpreting this result as further evolution could lead to increases in growth rate altering our model fits. It is also important to consider that fitness could be increasing in ways that are not captured by our growth rate estimates so that growth rate may have plateaued while fitness is still increasing in other ways, for example, increase in carrying capacity or decrease in lag time (Li et al., 2018).

### 3.2 Evolution at a higher temperature results in faster convergence among genotypes

To determine which factors affect the evolutionary trajectories, we fit a GAM (Figure 2a) and found that including the three‐way interaction between genotype, temperature, and generation produced the best fit based on the AICc score. Based on this result, we fit a standard least square model using the same terms and found that the scaled effect of the three‐way interaction of generations by slower growing parent (A) by 24°C was significantly negative (*p* < .0001), while the effect of generations by slower growing parent (A) by 37°C was significantly positive (*p* < .0001). This result indicates that genotypes are converging faster at the higher temperature. This can be seen in Figure 2a, where genotypes converge earlier and more fully at 37°C.

To further explore this result, we used *t*‐tests to determine at which 1,000‐generation bins there remains a difference between genotypes at each temperature (Figure 2b). A difference between genotypes persisted at both temperatures for a least 2,000 generations of evolution. However, after 2,000 generations the populations evolved at 37°C begin to converge and genotypes were no longer significantly different for the remainder of the experiment, while at 24°C there was a significant difference between two of the genotypes at 3,000 generations (Figure 2b). This trend remained into later generations, though the significance levels fail to meet the Bonferroni corrected threshold for most comparisons. During this time, the difference in the mean growth rate between the slower growing genotype (A) and the other two genotypes (A × B and B) is larger at the colder temperature, suggesting these populations fail to fully converge until the final time point at 6,000 generations, a full 2,000 generations longer than it takes populations evolved at 37°C (Figure 2b).

### 3.3 Temperature affects repeatability among populations

To test whether apparent differences between replicate populations evolved from a single ancestral individual were significant, we performed a nested ANOVA on *R*_P_ in the 6,000‐generation bin (generations 5,500–6,500). We found a significant effect of replicate population nested within genotype (*F*(16,76) = 3.47, *p* < .0001, Bonferroni corrected significance threshold = 0.01) indicating significant divergence between populations evolved from a single ancestor. Similar results were obtained for other time points. In fact, even in the earliest bin (generation 0–500) there is an effect of population nested within genotype (*F*(21,283) = 2.65, *p* = .0002, Bonferroni corrected significance threshold = 0.01), indicating that populations began to evolve measurable differences in growth rate early in their evolution.

**FIGURE 3:**
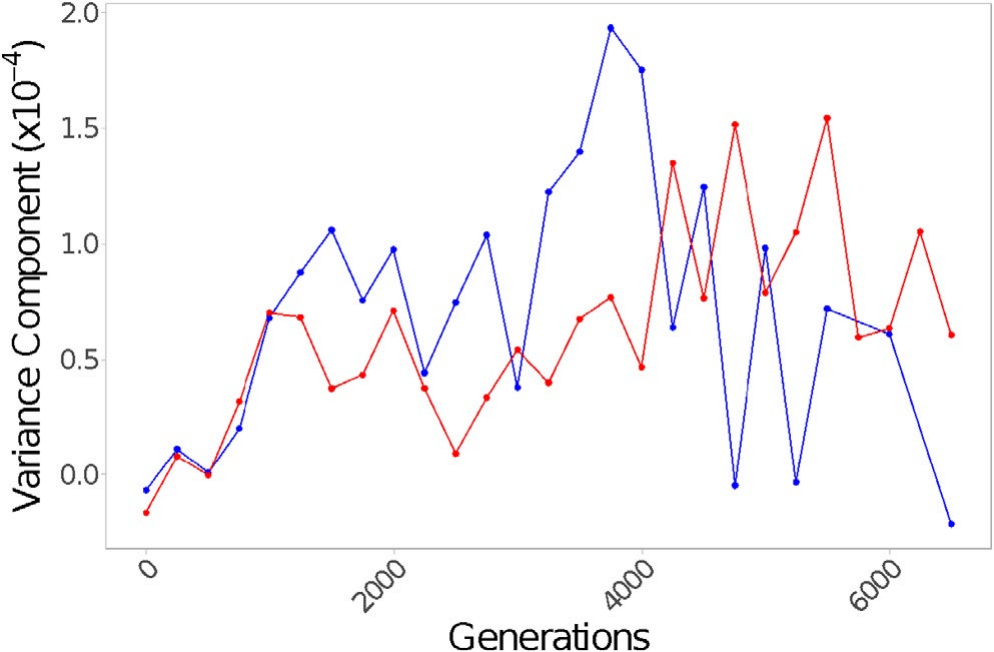
Variance in growth rate due to divergence among replicate populations. The variance components attributable to replicate population for populations evolved and assayed at 24°C (blue) or 37°C (red) over 6,500 generations of evolution. Variance components were estimated from an ANOVA on R_P_ with replicate population nested within genotype (*R*_P_ ~ *G* + *S*[*G*]) for each 250‐generation bin and evolution temperature

We consistently see a larger variance component attributable to replicate population nested within genotype among populations evolved and assayed at 24°C between generations 1,000 and 4,000 (Figure 3). This is true regardless of assay temperature (data not shown), indicating that evolution temperature is likely driving this effect. These results support our hypothesis that temperature impacts the repeatability of the growth rate trajectories of replicate populations.

**FIGURE 4:**
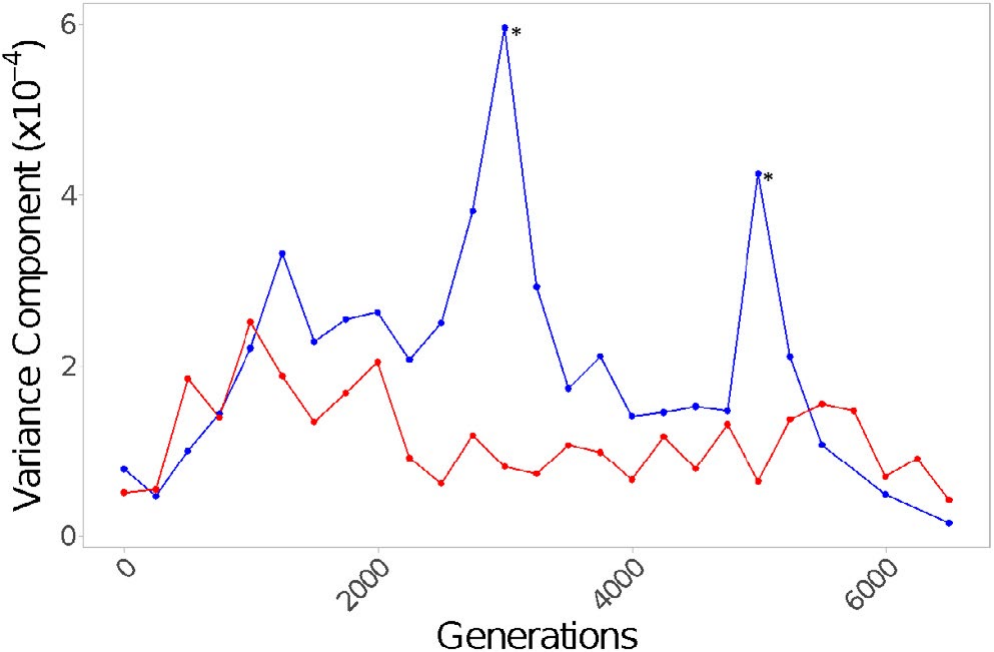
Variance in growth rate among all populations is lower for the hotter populations. The variance components attributable to population for populations evolved and assayed at 24°C (blue) or 37°C (red) over 6,500 generations of evolution. Variance components were estimated from an ANOVA on R_P_ without population nested within genotype (*R*_P_ ~ *S*) for each 250‐generation bin and evolution temperature. Asterisks indicate significant results of Levene's test

When we combine growth rate data from all genotypes, Levene's tests indicate there is a significant difference in the variance among populations at either temperature at generation 3,000 and generation 5,000 (Figure 4). We also find consistently lower variance components attributable to population among 37°C‐evolved populations than those evolved at 24°C (Figure 4). This is due to the joint effect of less divergence between replicate populations of the same genotype (Figure 3) and more convergence among different genotypes for populations evolved at 37°C relative to those evolved at 24°C (Figure 2). At both temperatures, the variance component attributable to population appears to peak at intermediate generations, although the peak is higher and later for populations evolved at 24°C, as variation accumulates among replicate populations but before genotypes have had sufficient time to converge (Figure 4).

In spite of the greater variation among replicate populations of the same genotype evolved at 24°C (Figure 3), we still detect greater differences among genotypes when evolution takes place at 24°C (Figure 2). This indicates that the observed differences among genotypes at 24°C versus 37°C (described in the section above) are not just due to higher variability among replicate populations at the lower temperatures, but also to longer lasting differences between genotypes. Additionally, the increased variance among lines evolved at the colder temperature is consistent when we look at the growth rate at the alternate temperature indicating this pattern is not the result of measurement differences between the two temperatures and is indeed the result of the evolution temperature.

### 3.4 Asymmetry of the correlated responses

**FIGURE 5:**
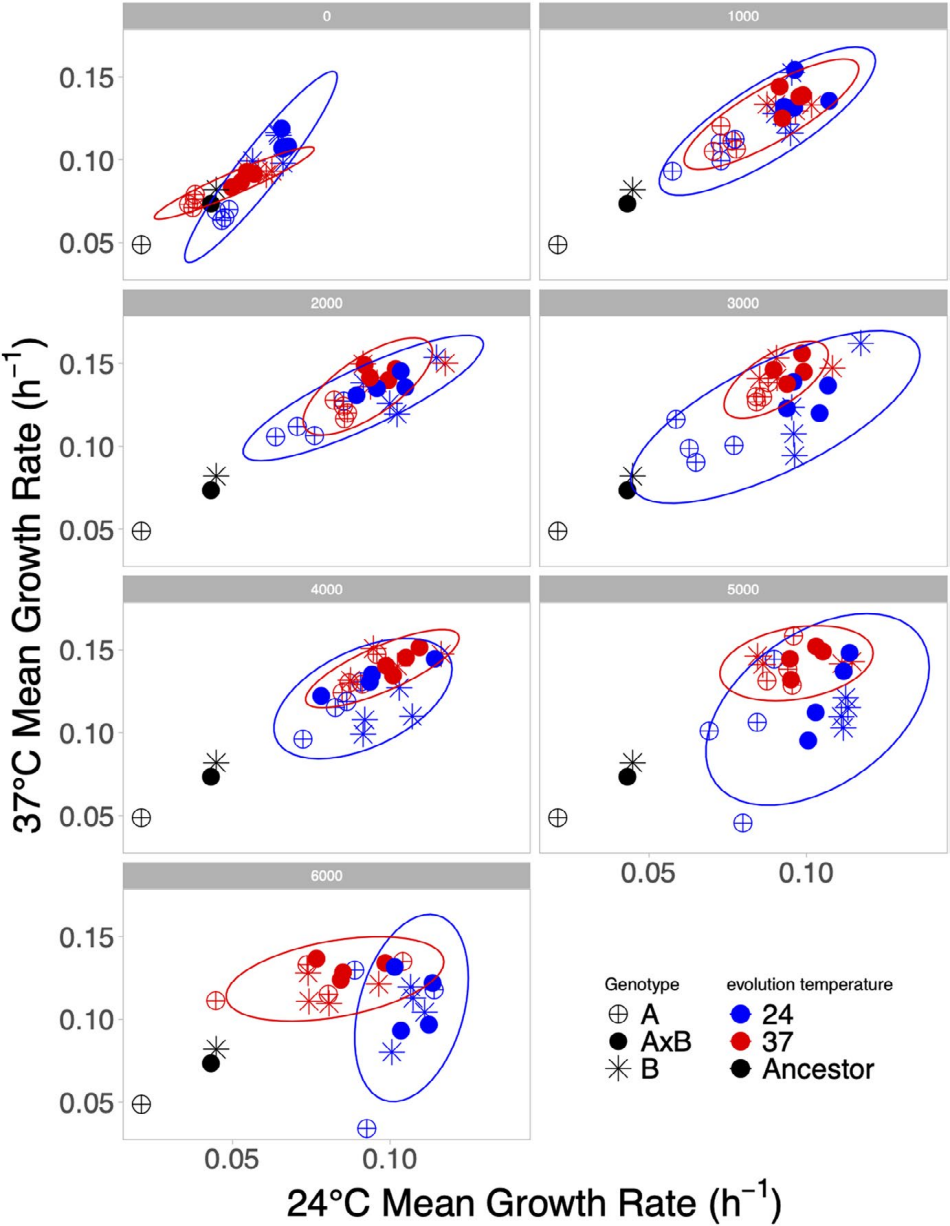
Correlation between growth rates in alternate environments. Growth rate of populations are shown every 1,000 generations measured at 37°C (*y*‐axis) or 24°C (*x*‐axis). Genotypes are indicated by symbols and the evolution environment is indicated by red (37°C) or blue (24°C) with ancestors shown in black. A trade‐off exists if an evolved population has lower fitness than its ancestor at the alternate temperature from which it evolved. While some populations have lower growth rates than the ancestor, in no case are these differences significant. The 95% confidence ellipse is shown for populations evolved at 37°C (red) and for populations evolved at 24°C (blue)

By generation 6,000, 21/24 populations significantly increased in growth rate at both the temperature in which they evolved and the alternate temperature (Figure 5), while no populations significantly decreased in growth rate indicating limited evidence of trade‐offs at this time point (*t*‐tests, Bonferroni corrected significance threshold = 0.00208). However, we find a significant interaction between evolution temperature and assay temperature at generation 6,000 (ANOVA on *R*_B_ at generation 6,000: *F*(1,37) = 18.6, *p* = .0001, Bonferroni corrected significance threshold = 0.00714), whereby populations evolved at 24°C tend to grow faster at 24°C and populations evolved at 37°C tend to grow faster at 37°C. This suggests that a portion of the adaptation that has taken place over the course of the experiment is temperature‐specific despite the lack of trade‐offs that we observe. Interestingly, this signature of temperature‐specific adaptation is not present in the first 2,000 generations of evolution (ANOVA on *R*_B_ at generation 2,000: *F*(1,38) = 1.73, *p* = .196, Bonferroni corrected significance threshold = 0.00714), indicating that the initial burst of adaptation at the start of the experiment is not temperature specific but instead likely general adaptation to the culture conditions shared across temperatures. The earlier time points show a stronger correlation between the growth rates at either temperature, which is gradually lost by the end of the experiment (Figure 5). Overall, our results suggest that there is an initial period of fast, mostly non‐temperature‐specific adaptation at both temperatures followed by slower non‐temperature‐specific adaptation at 37°C and slower temperature‐specific adaptation at 24°C (Figure 1). Therefore, continued evolution, particularly, at 24°C may eventually lead to more trade‐offs among these populations when assayed at the alternate temperature.

To further assess the asymmetry in the correlated response seen in Figure 1, we performed Tukey tests on the mean growth rates per population in each 1,000‐generation bin to test the effect of evolution temperature on the growth rate at either assay temperature. This helps identify which temperatures were driving the interaction between evolution temperature and assay temperature at each timepoint. At time points 3,000, 4,000, and 5,000, we found a significant effect of evolution temperature when assays were performed at 37°C (*R* ^2^ = 0.360, 0.425, and 0.408) but, remarkably, not at 24°C (*R* ^2^ = 0.0112, 0.0730, 0.0178; Tukey‐Kramer: *p* < .05). This means that even after 5,000 generations of evolution, the temperature at which populations evolved makes no difference when growth rate is assayed at 24°C. This supports the result in Figure 1 of a greater correlated response when evolution occurs at 37°C (e.g., populations evolved at 37°C show concomitant increases in growth rate at 24°C) and is consistent with the only populations showing indications of trade‐offs having evolved at the colder temperature (Figure 5).

## 4 DISCUSSION

We examined the evolutionary trajectories of populations of different genotypes of *T. thermophila* under differing temperature regimes. Our experimental design allowed us to test how evolution temperature affects repeatability, as well as how it impacts historical differences as evolution progressed at each temperature. We find that the higher evolution temperature led to more convergence among populations started from different genotypes, and less divergence among replicate populations of a single starting genotype, indicating that evolution at the higher temperature results in more repeatable fitness trajectories. Finally, we found asymmetry in the correlated responses, whereby evolution at the higher temperature increases fitness at the lower temperature more than the reverse, likely indicating more temperature‐specific adaptation at the lower temperature.

There are several important points to note in our results. First, evolutionary outcomes continue to change even after 1,000’s of generations; for example, some conclusions drawn from results after 4,000 generations are different after another 2,000 generations. Second, the factors that are important for predicting absolute increase in growth rate are different from those that are important for predicting the relative increase in growth rate. For example, while genotype is a significant predictor of both absolute and relative increase in growth rate, assay temperature is only a significant predictor of the relative increase in growth rate due to the different starting growth rates in each temperature. Finally, and importantly, due to the fact that populations grow faster at 37°C than 24°C, populations evolving at different temperatures experience differences in density, which may also contribute to the differences that we observe between temperature treatments.

### 4.1 Temperature affects the convergence of different genotypes

Over the course of evolution, different starting genotypes could converge in phenotype, evolve in parallel, or diverge even further, depending on factors such as epistasis, distribution of mutational effects, and strength of selection (Blount et al., 2018; Draghi & Plotkin, 2013; Kuzmin et al., 2018; Starr et al., 2018). Previous experiments have found that the rate of adaptation is inversely proportional to initial fitness and that initially different populations often end up at the same fitness optimum (Jerison et al., 2017; Wünsche et al., 2017). However, other studies have found that particular alleles can impede this fitness recovery and constrain the future of evolution (Jerison et al., 2017; Woods et al., 2011).

In our experiment, we observe the maintenance of historical differences between genotypes over many generations of evolution at both temperatures. Despite the overall increase in growth rate being greatest for the initially least fit genotype, we observe slower rates of adaptation for this genotype than we would expect if all genotypes followed the same pattern of diminishing returns. Further, temperature affects this pattern with differences between genotypes persisting much longer at 24°C than 37°C. Why a higher temperature would be more conducive to convergence is unclear but could be related to other effects of temperature observed in our experiment. For example, higher selection coefficients at 37°C could contribute to both the faster convergence and greater repeatability. Note, however, that the small number of genotypes used may have also contributed to this result limiting how generalizable it is across other species or even other *Tetrahymena* genotypes.

### 4.2 Temperature affects repeatability among populations

Previous studies have found differences in the repeatability of evolutionary trajectories under different environmental conditions (e.g., Bailey et al., 2015; Gresham et al., 2008). Here, we found that replicate populations of all genotypes diverged more at 24°C and were more repeatable at 37°C over many of the intermediate time points of the evolution experiment (Figure 4). This increased repeatability at 37°C is not present in the final time points of the experiment indicating the effect of temperature on repeatability may be transient, existing during periods of adaptation but disappearing in later generations.

Our observation of increased repeatability at 37°C could be explained by differences in the “ruggedness” of the fitness landscape, caused by epistatic interactions (Kvitek & Sherlock, 2011; Poelwijk et al., 2011). Alternatively, differences in repeatability could result from a difference in the distribution of beneficial mutations available in each environment (Lenski et al., 1991), in particular with beneficial mutations being more rare in the 24°C populations, resulting in greater differences between replicates. The strength of selection may also differ in these environments, and stronger selection is expected to result in greater repeatability (Bailey et al., 2017; Orr, 2005). Since 37°C is near the upper limit of the thermal tolerance for this species (Hallberg et al., 1985), populations at that temperature may experience greater selective pressure thereby causing the observed reduction in variation among populations evolved at this temperature.

### 4.3 Temperature affects correlated responses

Experiments using *E. coli* have found substantial evidence for temperature associated trade‐offs and asymmetry in correlated responses (Bennett & Lenski, 1993, 2007; Bennett et al., 1992; Mongold et al., 1996; Woods et al., 2006). In both *E. coli* and *T. thermophila*, evolution at a hotter temperature increases growth rate at a colder temperature, while evolution at a colder temperature increases growth rate at a hotter temperature less for *T. thermophila* and often decreases it for *E. coli* (Bennett & Lenski, 1993; Bennett et al., 1992; Mongold et al., 1996). The difference in our experiment is likely due, in part, to the fact that the ancestral genotypes were not well adapted to the general laboratory conditions (*T. thermophila* lines were derived from wild collected strains grown in laboratory ~500 generations before cryopreservation), whereas the *E. coli* experiments started from an ancestor that had already evolved under laboratory conditions for 2,000 generations. Over the first 2,000 generations of our experiment, changes in growth rate are positively correlated between evolution and alternate temperatures, indicating that populations are adapting more to the general culture conditions and not the specific temperature (Figure 1). This pattern continues for the entirety of the experiment for populations evolved at 37°C; however, populations evolving at 24°C begin adapting in a temperature‐specific manner after around 2,000 generations (Figure 1). After this, it still takes a further 4,000 generations of temperature‐specific adaptation at 24°C for just 3/12 populations to return to the ancestral growth rate at the alternate temperature. Further evolution in these environments is needed to determine whether trade‐offs will emerge.

The asymmetry we observe in the correlated responses could be due to the fact that as evolution occurs in one environment, fitness may change in other environments either due to pleiotropy or to the accumulation of mutations that are neutral in the evolution environment but have fitness consequences in the other environment (Cooper & Lenski, 2000). One possible mechanistic explanation for the observed asymmetry could be more transcript diversity, and thus more targets of selection, in hotter conditions if most genes that are transcribed at 24°C are also transcribed at 37°C but not vice versa. This would be consistent with the lack of antagonistic pleiotropy across temperatures among the most positively selected mutations found in laboratory‐evolved *E. coli* (Deatherage et al., 2017) and is supported by data showing that more genes are up‐regulated at hotter temperatures (Mittal et al., 2009; Tai et al., 2007).

Our findings of increased convergence and repeatability when evolution occurs at 37°C are consistent with the “hotter is better” hypothesis (Angilletta et al., 2010; Knies et al., 2009). However, this hypothesis does not explain the observed correlated responses of evolution in hotter conditions suggesting that different aspects of the 37°C environment may be responsible for greater convergence and repeatability, and the larger correlated response. In the future, more high‐throughput methods with greater control of the evolution conditions will allow for the identification of the precise environmental conditions responsible for the difference that we observed in evolution at different temperatures.

An alternative explanation for the difference in correlated responses is that populations evolving at 24°C adapt by increasing different components of fitness than those evolving at 37°C. We measured growth rate, which is a major component of fitness, and well correlated with competitive ability in our experiments, but fitness can also increase by, for example, decreasing lag time or increasing carrying capacity (Li et al., 2018). Future studies should take these traits into account.

A final caveat is that all of the adaptation that we observed occurred in the somatic nucleus, which is discarded following sexual reproduction. While there is evidence of some epigenetic inheritance between parental and progeny somatic genomes (Beisson & Sonneborn, 1965; Chalker & Yao, 1996; Pilling et al., 2017), it is unknown whether any of the adaptation that occurred in our experimental populations would be inherited by newly produced sexual progeny. However, this may be a moot point in this experiment because all of the evolved populations lost the ability to undergo sexual conjugation under our laboratory conditions.

## 5 CONCLUSION

One of the most important questions for evolutionary biologists is how variation builds up over time to create all of the diversity observed around us. Small incremental changes in isolated populations can, given enough time, lead to major differences in the organisms that make up those populations. However, selection can also result in striking examples of parallel and convergent evolution and we are only beginning to understand the ways in which genotype and the environment contribute to this process and to the overall repeatability of evolution. Here, we demonstrated that the temperature at which populations evolve can affect the patterns of evolution, with populations in hotter environments showing greater repeatability among replicates and faster convergence among genotypes. In addition, evolution at the hotter temperature results in populations that are more fit in the colder temperature than vice versa. These results support the growing body of work that demonstrate the importance of environment in determining evolutionary trajectories of populations. Further work using other species will be necessary to assess whether our findings with regard to temperature are generalizable or specific to *Tetrahymena*.

## CONFLICT OF INTEREST

The authors have no conflict of interest to report.

## AUTHOR CONTRIBUTIONS

**Jason Tarkington:** Conceptualization (equal); Data curation (lead); Formal analysis (lead); Investigation (lead); Methodology (lead); Resources (equal); Writing‐original draft (lead); Writing‐review & editing (equal). **Rebecca A. Zufall:** Conceptualization (equal); Writing‐review & editing (supporting).

### OPEN RESEARCH BADGES

This article has earned an Open Data Badge for making publicly available the digitally‐shareable data necessary to reproduce the reported results. The data is available at https://datadryad.org/stash/share/9VXlXdsKKBMAz‐LXxbpe0XgY7qBifoNXwjsqIDdsscw.

## Data Availability

Data are available on Dryad: https://doi.org/10.5061/dryad.31zcrjdm5.
